# Atypical Celiac Disease: From Recognizing to Managing

**DOI:** 10.1155/2012/637187

**Published:** 2012-07-03

**Authors:** B. Admou, L. Essaadouni, K. Krati, K. Zaher, M. Sbihi, L. Chabaa, B. Belaabidia, A. Alaoui-Yazidi

**Affiliations:** ^1^Laboratory of Immunology, Faculty of Medicine and University Hospital Center, BP 7010, Sidi Abbad, Marrakech, Morocco; ^2^Laboratory of Research “PCIM”, Faculty of Medicine, University Cadi Ayyad, Marrakech, Morocco; ^3^Service of Internal Medicine, University Hospital Center, Marrakech, Morocco; ^4^Service of Gastroenterology, University Hospital Center, Marrakech, Morocco; ^5^Service of Peadiatrics, University Hospital Center, Marrakech, Morocco; ^6^Laboratory of Biochemistry, University Hospital Center, Marrakech, Morocco; ^7^Laboratory of Histopathology, University Hospital Center, Marrakech, Morocco

## Abstract

The nonclassic clinical presentation of celiac disease (CD) becomes increasingly common in physician's daily practice, which requires an awareness of its many clinical faces with atypical, silent, and latent forms. Besides the common genetic background (HLA DQ2/DQ8) of the disease, other non-HLA genes are now notably reported with a probable association to atypical forms. The availability of high-sensitive and specific serologic tests such as antitissue transglutuminase, antiendomysium, and more recent antideamidated, gliadin peptide antibodies permits to efficiently uncover a large portion of the submerged CD iceberg, including individuals having conditions associated with a high risk of developing CD (type 1 diabetes, autoimmune diseases, Down syndrome, family history of CD, etc.), biologic abnormalities (iron deficiency anemia, abnormal transaminase levels, etc.), and extraintestinal symptoms (short stature, neuropsychiatric disorders, alopecia, dental enamel hypoplasia, recurrent aphtous stomatitis, etc.). Despite the therapeutic alternatives currently in developing, the strict adherence to a GFD remains the only effective and safe therapy for CD.

## 1. Introduction

Celiac disease (CD) is an intestinal chronic inflammatory and autoimmune disease that develops as a result of interplay between genetic, immunologic, and environmental factors [1]. Until recently, CD was considered to be a rare condition, with the highest incidence (1% to 0.3%) in European countries [2, 3]. The true incidence evaluated by a North American study is about 0.5% to 1%, but many, if not most, of studied patients were asymptomatic members of high-risk groups [3, 4]. Recent epidemiological studies performed in North Africa and *Asian* areas also showed a high rate of CD: 0.53% in Egypt [5], 0.79% in Libya [6], 0.6% in Tunisia [7], 0.88% in Iran [8], 0.6% in Turkey [9], and 0.7% in India [10]. The classic form of CD typically presents in infancy and manifests as failure to thrive, diarrhea, abdominal distention, developmental delay, and, occasionally, severe malnutrition [11, 12], which can lead to a true medical emergency [11]. Furthermore, serologic studies demonstrate that most celiac patients present with oligosymptomatic, latent, potential, and extraintestinal forms. These nonclassic clinical presentations become increasingly common and might reach about 50% of all diagnosed patients. The undiagnosed CD cases remain untreated, leaving individuals exposed to the risk of long-term complications, such as infertility, osteoporosis, or cancer [13–16].

Our aim is to emphasize the atypical clinical expression of celiac disease and suggest a diagnosis and managing approach.

## 2. Genetic Background

As demonstrated by several investigators, CD is one of the most common genetically based diseases; the part of genetic background is fundamental in its pathogenesis, with possible influence of genetic factors on clinical and immunologic features [17–19]. Approximately 97% of individuals with CD have genetic markers on chromosome 6p21, called class II human leukocyte antigen (HLA). HLA DQ2 predominates, occurring in 90–95% of patients, and HLADQ8 occurs in the remainder [11, 18, 20]. Some studies also point to a correlation between DQ2 homozygousness and female sex, earlier age at diagnosis, shorter time span between onset of symptoms and diagnosis, and to a higher prevalence of classic clinical presentations among patients carrying double-dose DQB1*02 [21]. Other investigations suggest that MHC class I region plays a role in the development of diverse clinical forms of the disease [19, 22]. López-Vázquez et al. [22], thus showed that haplotype B8/DR3/DQ2 is notably overrepresented in atypical CD patients compared to typical ones [19, 22]. In addition, similar studies displayed that MICA-A5.1 allele either is associated with atypical forms of CD in HLA-DQ2-negative patients or confers an additive effect to the DR3/DQ2 haplotype that may modulate the development of the disease [19, 23]. Also, linkage research pointed to chromosomal regions other than the HLA region, predisposing to CD with modest effects; the CTLA4 (cytotoxic T-lymphocyte associated), a closely located gene on chromosome 2q33, is one of these genes [1, 24]. Alongside the HLA, recent genetic studies concerning potential CD patients identified a robust association on chromosome 4q27, involving IL-2, IL-21, and KIAA1109 gene cluster [25, 26], and also c-REL gene [26]. These facts might allow more understanding in CD pathogenesis.

## 3. Clinical Faces of Celiac Disease

**Figure 1:**
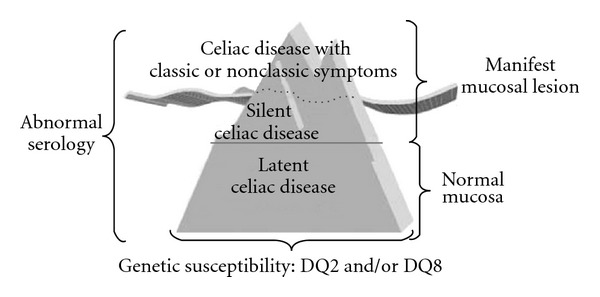
The celiac iceberg model [14].

Gee described the classical features of celiac disease in 1887 as diarrhea, lassitude, and failure to thrive [27], but the improvement of knowledge has subsequently disclosed several patterns of the disease [28]. A number of investigators believe that clinically apparent gluten-sensitive enteropathy represents the “tip of the iceberg” of the overall disease burden (Figure 1).

This concept demonstrates the clinical variability of CD and enlightens why the disease remains unidentified in a great proportion of individuals. In fact, the estimated ratio of diagnosed to undiagnosed individuals varies between 1 : 5 to 1 : 8 (the submerged part of the iceberg), usually because of atypical, minimal, or even absent complaints [13, 14].

Many authors defined atypical CD as follows:

- *Atypical form*. Absence or few gastrointestinal symptoms, presence of atypical symptoms, such as anemia due to iron deficiency, osteoporosis or osteopenia, infertility, low stature;
- *Silent form*. Occasional diagnosis, histological or serological, in asymptomatic individuals;
- *Latent form, with 2 categories*
  - patients with previous CD diagnosis who responded to gluten-free diet (GFD) and presented a normal histology or only intraepithelial lymphocytes increase,
  - individuals with normal intestinal mucosa, under diet including gluten, who will subsequently develop CD;
- *Refractory form*. Patients with CD who do not respond to GFD [12, 14, 29].

Patients with CD are diagnosed at any age and can exhibit a wide range of clinical manifestations (Table 1). In fact, beyond infancy, the symptoms of CD tend to be less dramatic [30, 31]. Older children may present with constitutional short stature or dental enamel defects, and women comprise approximately 75% of newly diagnosed adult CD cases, with more clinically conspicuous disease [11, 31].

Evidence suggests that the incidence of CD increases with age even in older patients [33]. Indeed, the majority of the elder cases remains undetected, often due to the absence of symptoms or because of atypical clinical presentations [34, 35]. Osteoporosis represents one of the most frequent revealing circumstances of the disease in the elderly, and the rate of bone loss is accelerated in women after the menopause, likewise in men at the same age [33, 36]. Anyway, physicians' lack of alertness in the older people may result in a significant delay in diagnosis, as CD is widely deemed to be a condition affecting younger subjects [33].

Moreover, a wider spectrum of neurologic syndromes may be the presenting extraintestinal manifestation of gluten sensitivity with or without intestinal pathology. These include headache, ataxia and psychiatric disorders [29], migraine, encephalopathy, chorea, brain stem dysfunction, myelopathy, mononeuritis multiplex, Guillain-Barré-like syndrome, and neuropathy with positive antiganglioside antibodies [37]. Additional studies showed high prevalence of gluten sensitivity in genetic neurodegenerative disorders such as hereditary spinocerebellar ataxia and Huntington's disease [37]. As well, oral manifestations, mostly recurrent apthous ulcers or stomatitis and dental enamel hypoplasia or defects, are atypical signs of CD, and should be considered, even in the absence of any gastrointestinal symptom, at-risk subjects, and should therefore undergo diagnostic procedure for CD [28, 38]. Also, recurrent febrile infections associated to moderate neutropenia must be included in the diagnostic workup for atypical/silent CD in the general population [39]. Furthermore, many of biologic abnormalities either concur with CD or at times may reveal the disease such as anemia with iron, vitamin B12 and/or folate deficiencies, hypertransaminasemia (Table 1).

The prevalence of CD has increased sharply in recent years because of better recognition of the disease and its associated disorders (Table 2) [18, 36, 40]. A number of diseases seem to occur more commonly in CD. Many studies showed that patients with type 1 diabetes mellitus (T1DM), autoimmune thyroid disease, Sjögren's syndrome, primary biliary cirrhosis, Addison's disease, systemic lupus erythematosus, and alopecia areata may also exhibit similar genotypes of the celiac disease (HLA-DQ2 [DQA1*0501 and DQB1*0201]) and are at risk for gluten-sensitive enteropathy [11]. Autoimmune disorders occur 3 to 10 times more frequently in those with celiac disease than in the general population. Evidence exists that the risk of developing other autoimmune conditions increases with length of exposure to gluten [11, 18, 41]. Among associated CD conditions, T1DM is probably the most important; occurring in about 5% of CD patients [40, 42], with a large variance between ethnic populations (range: 0.97–16.4%) [43]. In addition, unexplained and recurrent hypoglycemia in well managed diabetic individuals should alert the physician for CD screening [44]. Approximately 5% of the patients with CD have thyroid disorders (either autoimmune (Hashimoto's) or Graves's disease) [42], and the ISPAD (International Society for Pediatric and Adolescent Diabetes) clinical practice consensus guidelines 2006-2007 recommend an assessment of the thyroid function at the diagnosis of CD and thereafter every second year in asymptomatic individuals and also a screening for CD at time of the diagnosis of these thyroid disorders and every second year thereafter [45, 46]. Down or Turner syndromes also represent frequent linked conditions in which CD is often asymptomatic and then require systematic screening for CD [47, 48]. Furthermore, the association of some primary immunodeficiencies entities with CD has been described such as IgA deficiency [49] and common variable immunodeficiency [50].

## 4. Serologic Testing: Performances and Limits

**Figure 2:**
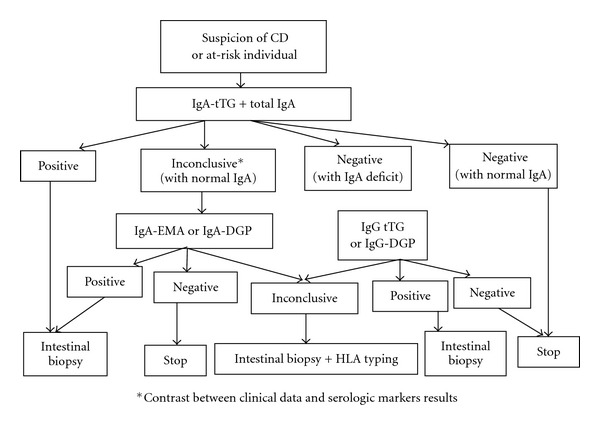
Algorithm proposal for biologic diagnosis of celiac disease.

Since the introduction of serological tests, and because of occasional screening, silent CD forms have been increasingly recognized. This is frequently the case of family predisposed individuals, and patients with associated autoimmune or genetic disorders. In CD, highly sensitive and specific methods are nowadays widely used in laboratory testing such as antiendomysial (EMA) and antitissue transglutaminase (tTG) antibodies tests [18, 51]. But, although these tests exhibit very high sensitivity and specificity [11, 13, 32], recent investigations showed that their accuracy remains controversial in some conditions; sensitivity is considered unacceptable both in patients with minor degrees of mucosal damage and in cases with silent or oligosymptomatic forms [32]. Moreover, EMA and tTG have been found to be superior to AGA (anti-gliadin antibodies) tests [11, 13, 18] and when used in combination have sensitivity and specificity greater than 95% [11, 13]. In addition, the recently developed deamidated gliadin peptide (DGP) antibody test shows promise in CD diagnostic [32, 51], and its performances are comparable to those of IgA-anti-tTG [52, 53]. Moreover, IgG anti-DPG test has high diagnostic sensitivity not only in IgA-competent but also in IgA deficient CD patients [52]. Therefore, a combined evaluation of IgA-anti-tTG, and IgG anti-DPG seems to be adequate for serodiagnosis of CD irrespective of IgA deficiency and without the need for estimating total IgA concentrations [52, 53]. The detection characteristics for AGA, EMA, tTG, and DGP tests are shown in Table 3. In practice, according to new recommendations, the initial serology testing consists on IgA-tTG screening, combined to total serum IgA measurement in order to rule out individuals with potential IgA deficiency. The serology test should be performed before eliminating gluten from patient's diet [54]. Actually, the biologic diagnosis should be improved by combining two performant serologic markers, such as IgA-tTG and -EMA or IgA-tTG and IgG-DGP according to suggested algorithm in Figure 2. The patients who test positive with these assays are consequently candidates for diagnostic endoscopy and small-bowel biopsy [51]. However, besides the atypical clinical expression of CD, the diagnosis may be more difficult for many reasons: negative serology, irregular histological behavior, or inadequate number or place of biopsies [55].

Despite the evolving performances of these serologic testing, there are still significant problems concerning the diagnosis approach in some atypical conditions; for example, it has been proposed that IgG-AGA testing might be the best marker for neurological manifestations of gluten sensitivity, mainly for patients with sporadic ataxia [60, 61]. Thus, in a recent study on gluten ataxia patients, Hadjivassiliou et al. [62], noticed anti-EMA antibodies in only 22% of patients, and anti-TG2 IgA in up to 38% of cases, but often at lower titres than those seen in patients with gluten sensitivity enteropathy [62]. On the other hand, the serology is generally thought to be unreliable in children <18 months of age [63]. This is due to a number of factors including the high proportion of children on breast milk, lower IgA levels, and the under-developed immune system. Some authors have suggested that IgA-AGA may be useful in this situation. This view is supported by a recent study carried out in 208 children <18 months of age diagnosed with CD [64], showing a better sensitivity of IgA-AGA compared to both the IgA-tTG and IgA EMA [65, 66].

## 5. Seronegative Celiac Disease

Not all patients have positive CD serologic markers at presentation [67, 68]. In fact, the presence of related CD antibodies correlates with the degree of villous atrophy and possibly the mode of presentation of the disease [67, 69]. Patients with lesser degrees of villous atrophy are less likely to have positive celiac serology [18], and patients who present persistently positive serology and negative biopsy probably have latent CD [12]. Moreover, children younger than 2 years of age lack EMA and tTG antibodies; for this reason, serological testing in children younger than 5 years of age may be less reliable and requires additional investigation [18]. On the other hand, in individuals who are IgA-deficient, the measurement of IgG-EMA and anti-tTG offers reliable results with excellent sensitivity (close to 100%) and specificity [12, 18]. Anyway, if CD suspicion is high with persistently negative tests, individuals must perform typing for HLA and, if positive, they must perform duodenal biopsy or alternatively perform biopsy directly [12, 55].

## 6. Histopathologic Findings

The intestinal biopsy represents the gold standard diagnosis for CD [12, 55]. According to Marsh-Oberhuber's [56, 57] criteria (Table 4), the spectrum of alterations compatible with CD consists of intraepithelial lymphocytic (IEL) infiltration, pattern of crypts, and villous atrophy, and patient's symptoms frequently correlate with the degree of tissue injury [59]. However, IEL increase with normal mucosa architecture may be observed in autoimmune diseases, such as SLE, rheumatoid arthritis, and Hashimoto's thyroiditis, in patients using nonhormonal anti-inflammatory treatment, in CD's initial presentation, and latent CD [55, 70]. An increase in IEL may also reflect a state of T cells activation triggered by gluten, immune abnormalities, drugs, and infectious agents. Celiac patients, who present only IEL increase with no alterations in the architecture of the mucosa, may be symptomatic and be under increased complications risk [12]. Similarly, villous atrophy may be due to other causes such as Crohn's disease, collagenous sprue, and autoimmune enteropathy [71]. Moreover, a recent prospective evaluation led by different expert pathologists highlighted that a recently proposed three-grade classification system [58] gives better interobserver agreement as compared with the established six-grade Marsh-Oberhuber classification (Table 4) [72].

Similarly to wide variation in clinical manifestations, GSE has a wide spectrum of histological abnormalities, which makes interpretation of small-intestinal biopsy specimens problematic for the pathologist [73]. Therefore, it is not advised to affirm a diagnosis based only on the histological findings, because the disease does not compromise uniformly intestine, and alterations are not observed exclusively in CD [12, 55]. Actually, many differential diagnoses (Table 5) may give rise to CD, making the diagnosis more difficult.

## 7. HLA Typing

All CD patients carry HLA-DQ2 or HLA-DQ8 [20]. However, up to 40% of the general population also carries these HLA haplotypes. Their presence is necessary for the development of celiac disease, but the absence of these alleles virtually excludes the diagnosis [18] with a negative predictive value for CD close to 100% [20]. HLA typing represents the first step for investigating relatives of CD patients, specifically 1st-degree relatives and then permits to identify individuals for evaluation with biopsy [12]. In practice, if CD suspicion is high, with persistently negative tests, individuals must perform typing for HLA and, if positive, they must perform duodenal biopsy or alternatively perform biopsy directly. Likewise, HLA typing is indicated in individuals who refuse to undergo biopsy [12].

## 8. Gluten-Free Diet: Indications and Managing

An increased incidence of small-bowel malignancies, adenocarcinoma, and enteropathy-associated T-cell lymphoma has been reported in untreated CD [18, 74].

A strict and lifelong gluten-free diet (GFD) has been demonstrated to be effective and safe, preventing most potential complications of the disease, including autoimmune disease, osteoporosis, infertility, prematurity, and malignancy [76, 77]. However, there is still no evidence that patients who have symptom-free celiac disease are at increased risk of small-intestinal lymphoma or other complications [71]. On the other hand, diet trials in patients with gluten sensitivity and neurologic syndromes have shown variable results and have been inconclusive in some neurologic diseases such as autism and schizophrenia [37]. Furthermore, in asymptomatic patients, a second follow-up biopsy under a GFD is advised to demonstrate the histological recovery of the mucosa, which usually does not develop before six months [73].

In general, the guidance of GFD may be envisaged according to three modalities (Table 6).

- Typical or symptomatic CD; GFD is a formal therapeutic indication.
- Silent CD; GFD is discussed under two circumstances.
- Silent CD discovered on the occasion of a serological screening in the family of a celiac or in a patient at risk (diabetes mellitus, dermatitis herpetiforme); in this case, the lesser clinical or nutritional sign would treat the subject as symptomatic and plead in favor of GFD.
- CD becoming silent in the second childhood after that the active disease in the first childhood was treated several years by a well monitoring GFD.

In these two situations and in individual really clinically and biologically asymptomatic, the decision to introduce or to resume the GFD is then rather preventive.

(v) Latent CD (subjects genetically predisposed with normal intestinal mucosa); a simple clinical and biological surveillance is advocated by recent studies [75].

Beside the GFD, the management of many of CD-linked features may require additional supplementation particularly in nutritional problems, such as lower Hb and low Fe, low albumin or Ca, cholesterol and folates disorders [32, 78–80]. Likewise, in CD patients with low bone mineral density, apart from a GFD, a rational managing should follow conventional lines, including increasing exercise, stopping smoking, and avoiding alcohol excess and ensuring an adequate Ca intake using supplements if necessary [36]. In addition, newly therapeutic alternatives are currently interested in the pathogenesis of the disease, focusing on engineering gluten-free grains, degradation of immunodominant gliadin peptides that resist intestinal proteases by exogenous endopeptidases, decrease in intestinal permeability by blockage of the epithelial zonuline receptor, inhibition of intestinal tTG2 activity by transglutaminase inhibitors, inhibition of gluten peptide presentation by HLA-DQ2 antagonists, modulation or inhibition of proinflammatory cytokines, and induction of oral tolerance to gluten [14, 81, 82]. But, at this time, strict adherence to a GFD remains the only effective and safe therapy for CD [14].

## 9. Refractory Celiac Disease

A small proportion of CD patients fails to improve after a GFD and may be considered as atypical regarding their outcome [14, 83]. Refractory celiac disease (RCD) was recently defined as persisting or recurring villous atrophy with crypt hyperplasia and increased intraepithelial lymphocytes (IELs) in spite of a strict GFD for more than 12 months [71, 84]. It can be either primary, as lack of initial response to diet, or secondary, as unresponsiveness to diet in the form of a relapse [73]. Two categories of RCD are recently being recognized: type I without aberrant T cells and type II with aberrant T cells [85]. The presence of an aberrant clonal intraepithelial T-cell population and/or loss of antigen on IELs seem to characterize population on high risk for development of overt lymphoma and differentiates RCD II from RCD I, which shows low or almost absent aberrant T cells [84].

To manage RCD, Krauss and Schuppan [71] recommend firstly to reassess the diagnosis of CD in order to exclude other diseases, such as giardiasis, tropical sprue, post-infectious diarrhea, collagenous sprue, protein intolerance or protein-losing enteropathy, tuberculosis (including atypical), AIDS, common variable immunodeficiency syndrome, Whipple's disease, ulcerative jejunitis, lymphocytic colitis, radiation enteritis, immunoproliferative small-intestinal disease, Crohn's disease, eosinophilic gastroenteritis, and autoimmune enteropathy [71, 84], and then to check for errors in diet or compliance [71]. The treatment of RCD I consists of a first-line immunosuppressive therapy based on azathioprine after induction of clinical remission with corticosteroids [86]. A second-line therapy (Cyclosporine A, infliximab, tacrolimus) is suggested in case of clinical deterioration despite corticosteroid therapy or intolerance to azathioprine [87] RCD II is usually resistant to medical therapies, and facing persistent clinical symptoms and/or a high percentage of aberrant T cells in intestinal biopsies in spite of a corticosteroid treatment, more aggressive therapeutic schemes should be considered [84].

## 10. Conclusion

Celiac disease represents a prototype of disease from which science and medicine take advantage, offering more and uninterrupted understandings both in genetic, clinic, diagnosis, and management aspects. Against its potential complications, the real challenge is to recognize asymptomatic or oligosymptomatic CD cases. The diagnosis should also be improved by a process of case finding focused on at-risk groups.

## Figures and Tables

**Table 1:** Clinical and biological revealing circumstances of atypical CD.

Atypical clinical symptoms	
Anemia	
Unclear vomiting	
Constipation	
Recurrent abdominal pain	
Short stature	
Irritability and impaired school performance	
Impaired physical fitness and chronic fatigue	
Osteopenia/osteoporosis/arthtritis	
Dermatitis herpetiformis	
Dental enamel hypoplasia	
Recurrent aphtous stomatitis	
Headache	
Peripheral neuropathy	
White matter lesions	
Cerebellar ataxia	
Epilepsy	
Intracranial calcifications	
Autism	
Psychiatric disorders	
Depression	
Pubertal delay	
Recurrent abortions	
Infertility	

Biologic abnormalities	

Anemia, iron deficiency; vitamin B12 and/or folate deficiency	
Hypertransaminasemia	
Hyperalkaline phosphatase level	
Hyperalbuminemia	
Hypercalcaemia, hypophosphatemia	
Thrombocytosis, leukocytosis	
Coagulopathy	
Low high-density and low-density lipoprotein cholesterol levels	

**Table 2:** List of possible celiac-disease-linked pathologies.

Associated autoimmune diseases or other conditions	
Type 1 diabetes	
Thyroid disorders (autoimmune or graves)	
Liver disease (autoimmune hepatitis, primary biliary cirrhosis)	
Myasthenia gravis	
Primary biliary cirrhosis	
Primary sclerosing cholangitis	
Psoriasis	
Sjögren disease	
Systemic lupus erythematosus	
Idiopathic dilated cardiomyopathy	
Immunoglobulin A nephropathy	
Lymphocytic or microscopic colitis	
Autoimmune Addison's disease	
Rheumatoid arthritis	
Vitiligo or alopecia areata	

Associated genetic diseases	

Down syndrome	
Turner syndrome	
Williams syndrome	
IgA deficiency	
Commun variable immunodeficiency	

**Table 3:** Characteristics of exclusive or combined serological tests used to detect CD [11, 13, 18, 32].

Serological tests	Sensitivity (%)	Specificity (%)	PPV (%)	NPV (%)
IgG AGA	57–78	71–87	20–90	40–90
IgA AGA	55–100	65–100	30–100	70–100
IgA EMA	86–100	98–100	98–100	80–95
IgA tTG	90–96	91–97	>90	>95
IgA tTG and EMA	98–100	98–100	>90	>95
IgA DGP	98	94	92	98
IgG DGP	97	100	100	97
IgA DGP + IgA tTG	100	93	91	100
IgG DGP + IgA tTG	100	97	97	100

IgG: immunoglobulin G; IgA: immunoglobulin A; AGA: antigliadin antibodies; EMA: endomysial antibodies; tTG: tissue transglutaminase; DGP: deamidated gliadin peptide; PPV: positive predictive value; NPV: negative predictive value.

**Table 4:** Histopathologic classification of CD based on Marsh-Oberhuber [56, 57], and Corazza and Villanacci [58] new grading system [12, 57, 59].

Marsh-Oberhuber classification	
(i) Marsh I: infiltrative lesion, normal villous architecture and mucosa, and IEL increase (>30–40 lymphocytes/enterocytes counted).	
(ii) Marsh II: hyperplasic lesion; similar to Marsh I with crypt hyperplasia.	
(iii) Marsh III: destructive lesion, subdivided to the following:	
(a) partial villous atrophy,	
(b) subtotal villous atrophy,	
(c) total villous atrophy.	

New grading system	

(i) Grade A (nonatrophic): >25 IELs/100 enterocytes.	
(ii) Grade B (atrophic): villous-crypt ratio <3 : 1.	
(iii) Grade B2 (atrophic): no detectable villi.	

**Table 5:** Celiac disease differential diagnosis [12].

Anorexia nervosa	
Autoimmune enteropathy	
Bacterial overgrowth	
Collagenous sprue	
Crohn's disease	
Giardiasis	
HIV enteropathy	
Hipogammaglobulinemia	
Gastroenterite infecciosa	
Intestinal lymphoma	
Radiation enteritis	
Ischemic enteritis	
Lactose intolerance	
Common variable immunodeficiency	
Soy protein intolerance	
Tropical sprue	
Tuberculosis	
Whipple's disease	
Zolliger-Ellison syndrome	
Eosinophilic gastroenteritis	

**Table 6:** Indications of GFD in CD of children and adolescents [75].

CD clinical form	Indications of GFD
Symptomatic	Therapeutic
Silent	Preventive: may be discussed
Latent	Surveillance
